# Beef cows with atypical estrous cyclicity at puberty produced calves with deficits in preweaning muscling, metabolic indicators, and myoblast function but not in feedlot performance^1^

**DOI:** 10.1093/tas/txaa119

**Published:** 2020-12-22

**Authors:** Rebecca M Swanson, Rachel L Gibbs, Caitlin N Cadaret, Galen E Erickson, Ty B Schmidt, Andrea S Cupp, Dustin T Yates

**Affiliations:** Department of Animal Science, University of Nebraska-Lincoln, NE

## INTRODUCTION

In cattle, age at puberty and number of estrous cycles prior to first breeding contribute to lifetime reproductive success (Perry et al., 1991). In our university beef herd, we have identified a subset of cows that exhibited irregular pubertal cyclicity patterns between weaning and their first breeding season, which we postulate is associated with high androstenedione in follicular fluid (Cupp et al., 2019). Cows with high androstenedione are subfertile but wean calves that average 17 kg heavier than the herd average (Summers et al., 2014). We hypothesized that this additional weight at weaning in their offspring is due to superior muscling and growth efficiency, characterized by better myoblast function, lean mass, and metabolic efficiency. The objective of this study was to test this hypothesis by evaluating growth and metabolic parameters in calves prior to weaning and in the feedlot, as well as carcass characteristics at harvest. We compared calves from cows that were classified as having typical pubertal cyclicity, start–stop pubertal cyclicity, or noncyclic puberty.

## MATERIALS AND METHODS

This study was approved by the Institutional Animal Care and Use Committee at the University of Nebraska-Lincoln, which is accredited by Association for Assessment and Accreditation of Laboratory Animal Care International. Steer calves were selected from crossbred Red Angus dams that had been previously classified into groups based on pubertal cyclicity pattern exhibited between weaning and their first breeding season (Cupp et al., 2019). Progesterone concentrations were determined in weekly blood samples over this period, and heifers were considered pubertal when concentrations were greater than 1 ng/mL. Heifers achieving and maintaining this progesterone concentration were classified as having typical pubertal cyclicity. Heifers reaching this concentration but subsequently falling below were classified as having start–stop pubertal cyclicity. Heifers not reaching this concentration were classified as being noncyclic at puberty. In two consecutive years, steer calves were randomly selected from each of the three maternal pubertal groups (*n* = 10/group/yr) to evaluate offspring growth and performance. Blood samples and muscle biopsies were collected at 3 mo of age and calves were weaned at 7 mo. After weaning, calves were fed ad libitum corn-based diets over an 85-d growing period followed by a 168-d finishing period. Steers were then harvested at a commercial abattoir.

Blood was collected via jugular venipuncture and plasma glucose concentrations were measured via glucose meter (One Touch). Plasma insulin was determined with a commercial Enzyme-linked aminoassay kit (Alpco). Blood plasma urea nitrogen, cholesterol, high-density lipoprotein-cholesterol, and triglycerides were determined with a Vitros-250 Chemistry Analyzer (Ortho). Biological impedance analysis was performed to estimate body composition as previously described (Gibbs et al., 2019).

Primary myoblasts were isolated from semitendinosus muscle biopsies as previously described (Yates et al., 2014). Ex vivo proliferation rates were determined during a 2-h EdU pulse after growing myoblasts in complete growth media for 3 d (Posont et al., 2018). Media contained 0 or 10 μm androstenedione (Sigma) for 24 h prior to and during the pulse. Myoblasts replicating during the 2-h period were identified using the Click-iT 5-Ethynyl-2′-deoxyuridine staining kit (ThermoFisher) and counted via flow cytometry (Orflo).

Oxidative and glycolytic metabolism in primary myoblasts were determined via a Seahorse XF Mito Stress Test (Aligent). Myoblasts from each calf were incubated with 0 or 5 mU/mL insulin (Ely Lilly). To estimate oxidative metabolism, oxygen consumption rates (OCR) under baseline conditions and for proton leak, maximal respiration, nonmitochondrial respiration, respiration for adenosine triphosphate (ATP) production, coupling efficiency, and spare respiratory capacity were measured via sequential injection of 1 µM oligomycin, 1 µM Carbonyl cyanide-p-trifluoromethoxyphenylhydrazone, and 0.5 µM rotenone. To estimate glycolytic metabolism, extracellular acidification rates for nonglycolytic acidification, glycolysis, and glycolytic capacity were measured during the same injections.

Growth data, blood components, and bioelectrical impedance data were analyzed by analysis of variance using the mixed procedure of SAS. Fisher’s least significant difference was used for mean separation. Data from myoblast studies were analyzed with incubation condition as a repeated measure. For myoblast studies, three technical reps/media condition were performed and averaged. Calf is the experimental unit, and data are presented as mean ± SE.

## RESULTS

**Figure 1.**
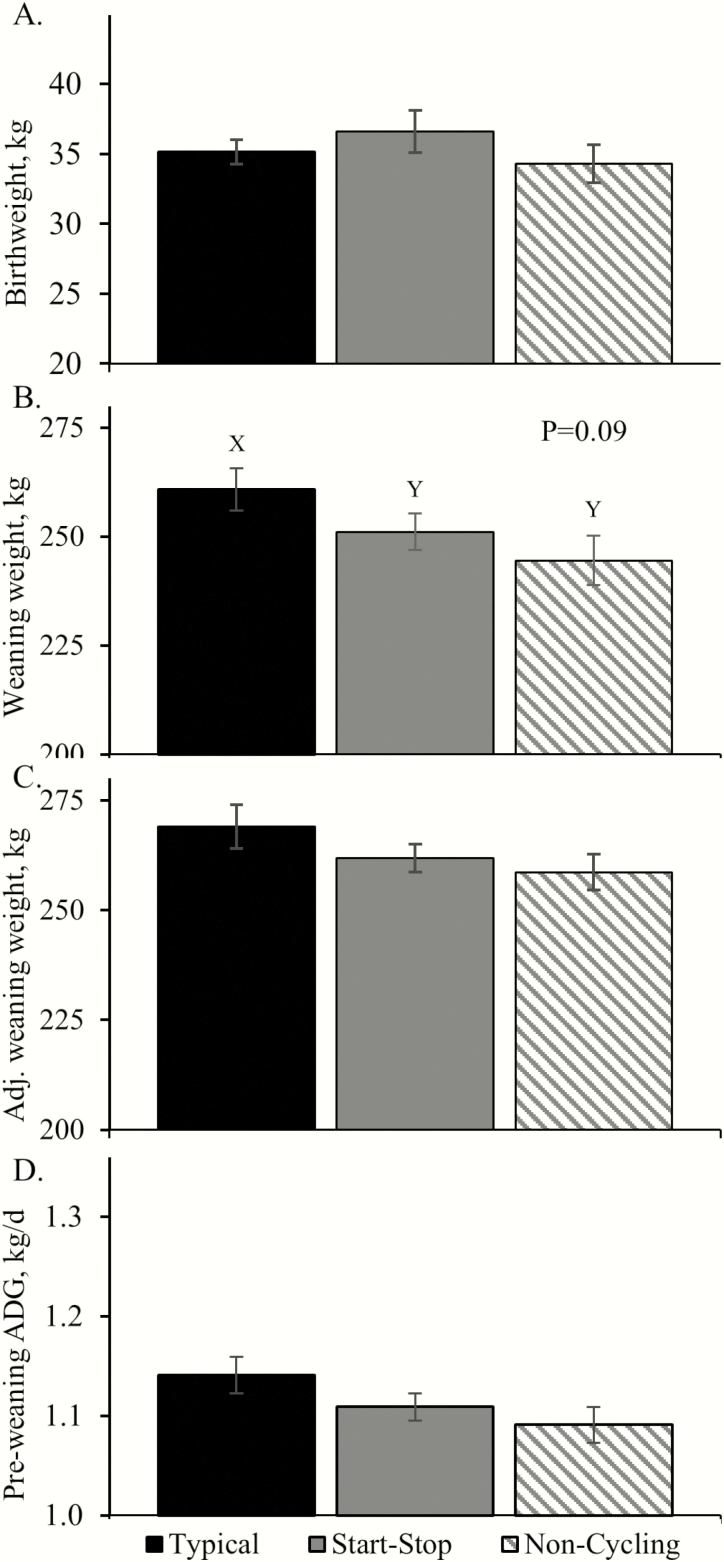
Early growth in calves from cows that exhibited irregular (start–stop) or absent (noncycling) estrous cyclicity at puberty. ^X,Y^Means with differing superscripts tend to differ (*P* < 0.10).

Cows with noncyclic or start–stop pubertal cyclicity calved 11.0 and 6.8 d later (*P* < 0.05) in the calving season, respectively, than cows with typical pubertal cyclicity, but birthweights did not differ among groups (Fig. 1). Weaning weight tended to be reduced (*P* = 0.09) in calves of cows with start–stop and noncyclic pubertal estrous compared to typical pubertal estrous, but 205-d adjusted weaning weights did not differ. At 3 mo of age, fat-free lean mass and soft tissues estimated by bioelectrical impedance tended to be decreased (*P* = 0.10) in calves from cows with start–stop and noncycling compared to typical pubertal estrous. The estimated sums of hind-quarter muscles only, of all muscles, and of all muscles with lean trim each tended to be decreased (*P* = 0.10) in calves from cows with start–stop and noncycling compared to typical pubertal estrous. Estimated moisture, protein, and fat content did not differ among groups.

**Figure 2.**
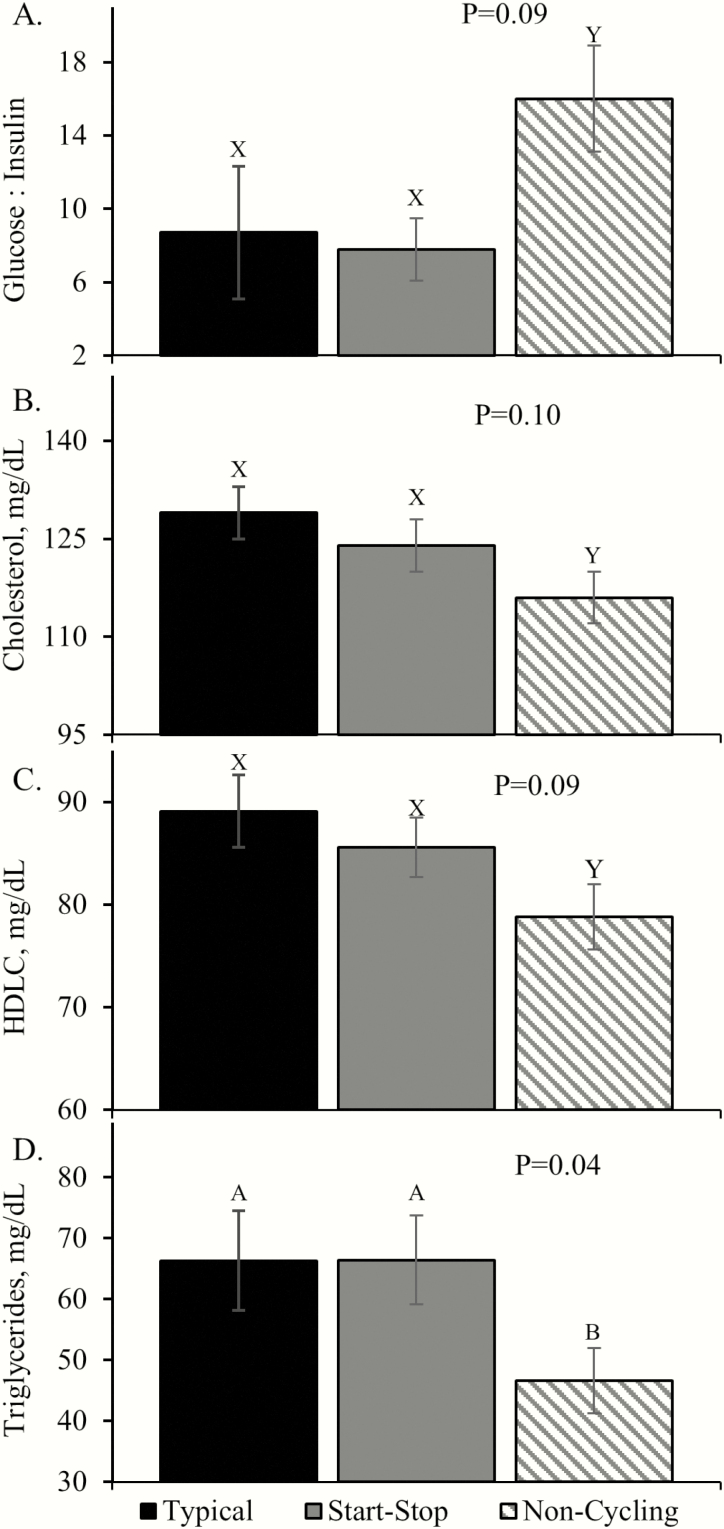
Early metabolic parameters in calves from cows that exhibited irregular (start–stop) or absent (noncycling) estrous cyclicity at puberty. ^A,B^Means with differing superscripts differ (*P* < 0.05). ^X,Y^Means with differing superscripts tend to differ (*P* < 0.10).

Plasma glucose and insulin concentrations did not differ among groups at 3 mo of age, but glucose-to-insulin ratios tended to be increased (*P* = 0.09) in calves from cows with noncycling pubertal estrous (Fig. 2). Plasma urea nitrogen did not differ, but plasma cholesterol and high-density lipoprotein cholesterol tended to be decreased (*P* = 0.09) and triglycerides were decreased (*P* < 0.05) in calves from cows with noncycling pubertal estrous.

**Figure 3.**
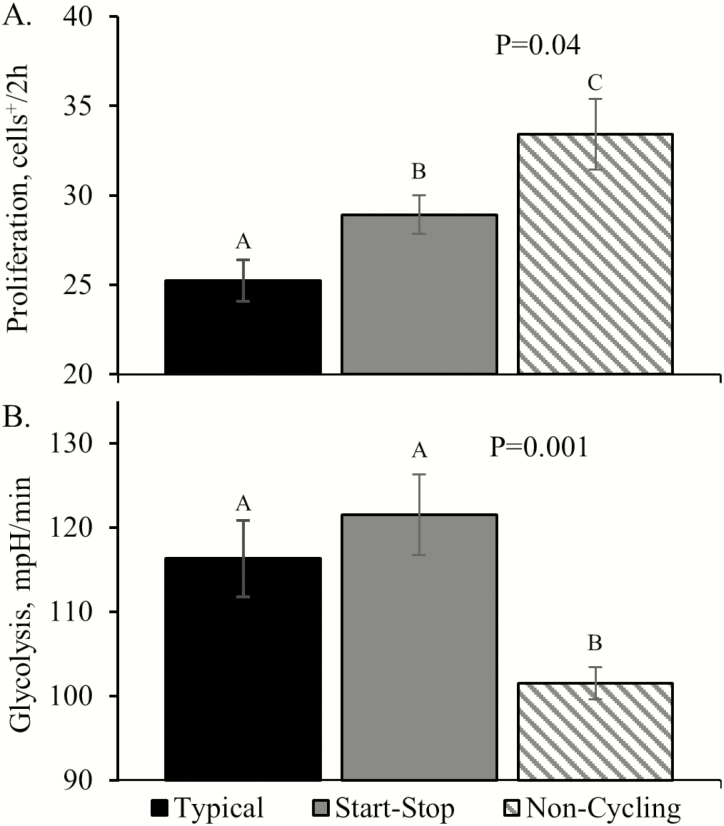
Proliferation and glycolytic rates in primary myoblasts of calves from cows that exhibited irregular (start–stop) or absent (noncycling) estrous cyclicity at puberty. ^A,B,C^Means with differing superscripts differ (*P* < 0.05).

Proliferation rates were increased (*P* < 0.05) in myoblasts isolated from the calves of start–stop and noncycling cows compared to typical cycling cows but did not differ between basal and androstenedione-spiked media (Fig. 3). Baseline respiration and proton leak rates of myoblasts did not differ among groups. Maximum respiration rates were increased (*P* < 0.05) and maximum respiration/total OCR tended to be increased (*P* = 0.07) in myoblasts from calves of noncycling cows. Spare respiratory capacity was decreased (*P* < 0.05) in myoblasts from calves of start–stop cows and spare respiratory capacity/total OCR tended to be increased (*P* = 0.07) in calves of noncycling cows. Nonmitochondrial OCR, oxygen consumption for ATP production, and coupling efficiency did not differ among groups. Nonglycolytic acidification did not differ, but glycolysis rates and glycolytic capacity were reduced (*P* < 0.05) in myoblasts from calves of noncycling cows. Glycolysis rates were increased (*P* < 0.05) in myoblasts incubated in insulin-spiked media compared to basal media, regardless of maternal group.

**Figure 4.**
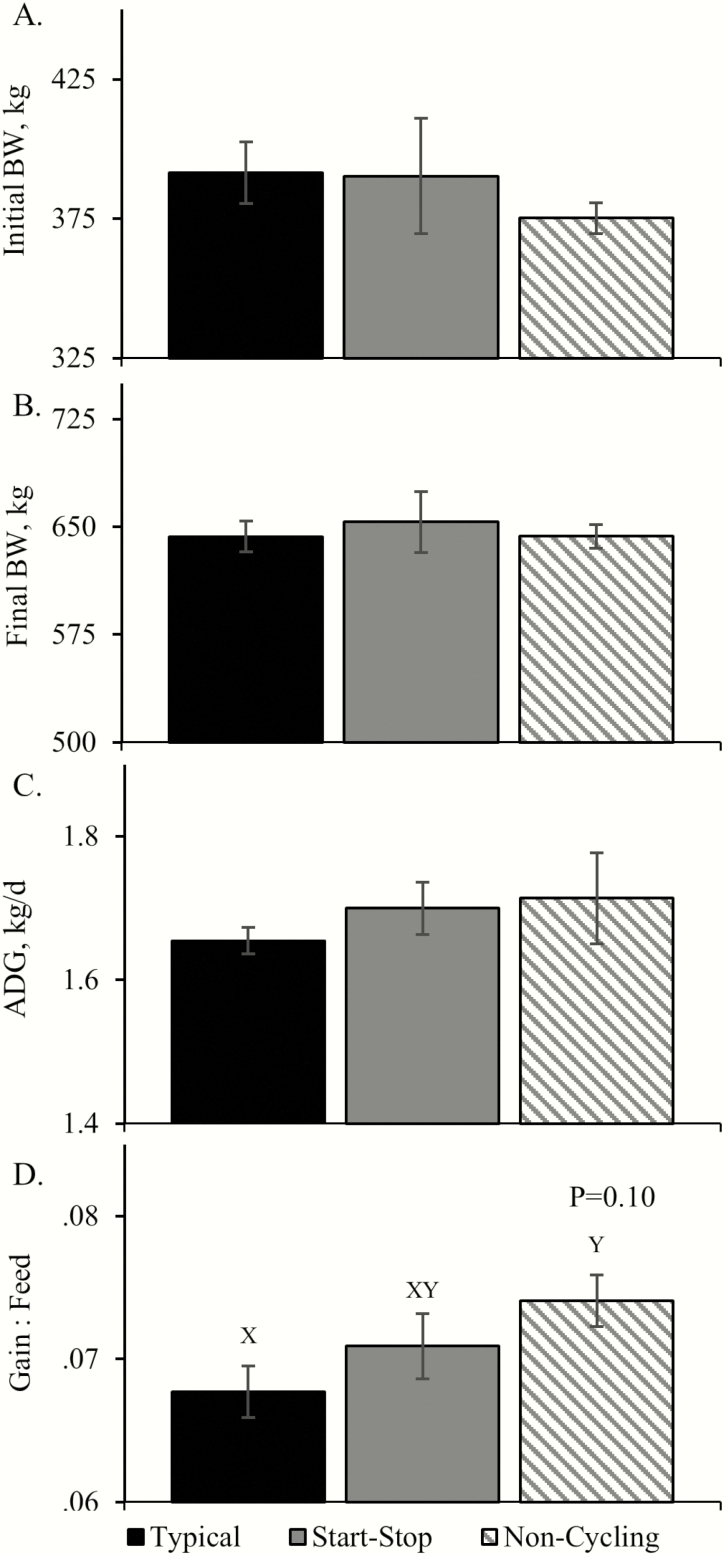
Feedlot performance metrics in calves from cows that exhibited irregular (start–stop) or absent (noncycling) estrous cyclicity at puberty. ^X,Y^Means with differing superscripts tend to differ (*P* < 0.10).

Initial and final body weight, average daily gain, and dry matter intake in the growing and finishing phases of the feedlot did not differ, but feed efficiency tended to be increased (*P* = 0.10) in calves from cows with noncycling compared to typical pubertal estrous in both phases (Fig. 4). However, carcass-adjusted final live body weight, average daily gain, and feed efficiency did not differ among groups. At harvest, dressing percentages, hot carcass weights, ribeye areas, 12th-rib fat thicknesses, and marbling scores did not differ among groups.

## DISCUSSION

In this study, we found that irregular and delayed reproductive cyclicity from weaning to first breeding in heifers did not affect subsequent growth rates of their offspring preweaning or in the feedlot. However, calves born to cows expressing these irregularities had reduced estimated muscling prior to weaning. We speculate that this reduction was associated with greater inflammatory regulation, which our previous findings in sheep indicate decreases muscle growth and increases fat deposition (Gibbs et al., 2019). Moreover, myoblasts isolated from these calves had increased proliferation rates but decreased glycolytic capacity, which our previous findings suggest could also reflect heightened inflammatory regulation (Cadaret et al., 2017; Posont et al., 2018). Greater proliferation can be indicative of an inability of myoblasts to exit the cell cycle and differentiate (Yang et al., 2012; Contreras et al., 2018), which is a necessary step for muscle growth. Similar adjusted 205-d weaning weights combined with indicators of diminished muscle mass suggest that body composition is affected in calves from cows with irregular or delayed cyclicity. Moreover, the observed differences in actual (i.e., unadjusted) weaning weights were likely due to the later calving dates for cows with atypical pubertal cyclicity, as their poor fertility (Summers et al., 2014; Cupp et al., 2019) presumably led them to become pregnant later in the breeding season in this study. Surprisingly, we observed no differences in growth traits among calves during the feeding period even when adjusted for carcass metrics. This perhaps indicates a capacity for catch-up growth in calves of cows with irregular or delayed cyclicity, although it is not clear from this study what the mechanism might be. We recognize that additional experimental units might clarify our findings for the feedlot phase and that this is a limitation of this study. This is particularly true considering that enhanced cellular replication coupled with indicators of impaired glucose metabolism in muscle stem cells from calves of cows with abhorrent early cyclicity would indicate poor functional efficiency prior to entering the feedlot. Nevertheless, these findings show that delayed puberty and atypical reproductive cyclicity patterns of beef cows between weaning and their first breeding season coincided with unusual myoblast function and metabolism, as well as altered body composition in their offspring but not in preweaning rates of gain, feedlot performance, or carcass merit.

## IMPLICATIONS

These data confirm an association between atypical maternal cyclicity at puberty and early metabolic changes in offspring that coincided with indicators of reduced muscle mass. Although the underlying cause is unclear, many of our findings would be consistent with heightened inflammatory activity in calves from cows that exhibited irregular cyclicity between weaning and their first breeding season. Thus, there is a clear need to further investigate the potential role of inflammation and to identify possible mediators that link abhorrent pubertal cyclicity in dams to these conditions in their offspring. Identifying the mechanisms for this link, inflammation or otherwise, will allow for management strategies to address early muscle growth and metabolic deficits in these calves.
